# Protective Effects of Fullerene C_60_ Nanoparticles and Virgin Olive Oil against Genotoxicity Induced by Cyclophosphamide in Rats

**DOI:** 10.1155/2018/1261356

**Published:** 2018-07-15

**Authors:** Fayza M. Aly, Amnah Othman, Mohie A. M. Haridy

**Affiliations:** ^1^Department of Zoology, Faculty of Science, South Valley University, Qena 83523, Egypt; ^2^Leibniz-Institut für Analytische Wissenschaften-ISAS-e.V., Bunsen-Kirchhoff-Straße 11, 44139 Dortmund, Germany; ^3^Department of Pathology & Clinical Pathology, Faculty of Veterinary Medicine, South Valley University, Qena 83523, Egypt

## Abstract

The potential effects of the fullerene C_60_ nanoparticle (C_60_) as well as virgin olive oil (VOO) against the cyclophosphamide- (CP-) induced cytotoxic and mutagenic effects were evaluated by two main methods: molecular intersimple sequence repeat (ISSR) assay and cytogenetic biomarkers. Thirty adult male rats were divided to five groups (control, CP, C_60_, CP + C_60_, and CP + VOO). CP was i.p. injected with a single dose of 200 mg/kg; C_60_ and VOO were given orally (4 mg/kg dissolved in VOO and 1 ml, resp.) in alternative days for 20 days. The ISSR analysis revealed an increased in the DNA fragmentation level for liver and heart tissues represented by 21.2% and 32.6%, respectively, in the CP group. The DNA polymorphism levels were modulated and improved in CP + C_60_ (8.9% and 12%) and CP + VOO (9.8% and 12.7%) for hepatic and cardiac tissues, respectively. The bone marrow cytogenetic analysis revealed that C_60_ and VOO had significantly decreased the frequency of CP-induced chromosomal aberrations (chromosomal ring, deletion, dicentric chromosome, fragmentation, and polyploidy). Fullerene C_60_ and VOO have ability to reduce DNA damage and decrease chromosomal aberrations. In conclusion, fullerene C_60_ and VOO have protective effects against the CP-induced mutagenicity and genotoxicity. Fullerene C_60_ and VOO open an interesting field concerning their potential antigenotoxic agents against deleterious side effects of chemotherapeutics.

## 1. Introduction

Commonly used anticancer agents, for example, cyclophosphamide, are implicated as mutagenic agents against mammalian cells *in vivo* and *in vitro* [1, 2]. Cyclophosphamide causes cytotoxicity to normal cells in spite of its effective anticancer alkylating agent [3]. The active metabolites of cyclophosphamide, for example, phosphoramide mustard and acrolein, are responsible for accumulation of reactive oxygen species resulting in fragmentation of the DNA strand and an increasing in mutagenic DNA effects [4, 5]. The activated CP metabolites are responsible for inducing damage to DNA, RNA, proteins, and cytoplasmic membranes [6, 7]. Therefore, it is necessary to investigate an effective antioxidant that prevents the oxidative DNA damage and reduces the side effects of CP and other chemotherapeutic agents.

Recently, carbon nanotubes, especially fullerene, have received considerable attention in the field of biomedical research and applications due to their distinct electrical properties. The interactions between carbon nanotubes, proteins, nucleic acids, and cell membranes as well as their mutagenicity and antimutagenicity assays have been investigated in order to discover potential antimutagen and anticarcinogen potentials [8]. Evaluation of chromosomal aberration is an effective assay to detect the occurrence of the genotoxicity. Detection of chromosomal aberration is an indicator for an organism exposure to the genotoxic agent and the occurrence of DNA damage. Various types of mutagens can induce structural chromosomal aberrations via DNA strand breaks that may elevate the risk of developing tumors [9, 10]. It is necessary to approve potential drugs that can be used in protection and amelioration of cytotoxicity and DNA damage. The genotoxic effect of fullerene C_60_ (C_60_) is controversial. C_60_ has genotoxic activity resulting in breaks of the DNA strand as well as oxidative damages of DNA in a concentration-dependent manner. The basic mechanisms of its toxic effect are lipid peroxidation, oxidative stress dissemination, and genotoxicity [11–13]. It was found that С_60_ toxicity depends on their surface modifications, synthesis, concentration in the medium, and processing conditions. On the other hand, numerous studies found no mutagenic effect of C_60_ fullerene *in vivo* and *in vitro* [14–17]. C_60_ fullerene nanoparticle does not possess any genotoxic effect towards human lymphocytes. C_60_ was used in combination with doxorubicin (one of the most common anticancer therapeutic agents); C_60_ reduced the genotoxic effect of doxorubicin in healthy human lymphocytes [18]. Furthermore, С_60_ possesses an ability to prevent oxidative stress dissemination due to the nanosize [19, 20].

Olive oil-containing meals reduce the risk of many diseases and malignant tumors, as they have antioxidative, anti-inflammatory, and anticarcinogenic effects [21]. Oleuropein and hydroxytyrosol are important components of virgin olive oil (VOO); they have anticancer activity through reducing DNA oxidation, arresting the cell cycle, and inducing apoptosis in tumor cells [22]. High consumption of VOO in the Mediterranean diet has been suggested to be responsible for protection of DNA against perioxidation and hence reduction in cancer incidence. So, it was found that DNA and RNA oxidation in Northern European regions is higher compared with that in central and Southern regions. These findings support the assumption that VOO consumption may explain the decreased incidence of cancer in south European than those in North regions [23]. It is necessary to investigate the effect of C_60_ nanoparticle and VOO separately and in combination against CP-induced genotoxicity. Therefore, the objective of this study is to assess the effect of C_60_ as well as VOO on CP-induced genotoxicity in rats based on detection of DNA damage by intersimple sequence repeat (ISSR) analysis of liver and heart tissues and detection of chromosomal aberrations in bone marrow cells by mitotic analysis technique.

## 2. Materials and Methods

### 2.1. Animals, Experimental Design, and Sampling

Thirty male albino rats (weighing 180–200 g, 2-month old) were housed in Animal House Facility (South Valley University, Qena, Egypt). Rats were housed under normal nutritional and laboratory conditions for one week for acclimatization. Animals were kept in the ventilated room under controlled laboratory conditions of normal light-dark cycle (12 h light/dark) and temperature (25 ± 2°C). Food and water were provided ad libitum. Rats were divided into 5 groups (*n* = 6 rats each group) as follows: (i) control group received placebo intraperitoneal (i.p.) injection; (ii) CP group was injected i.p. with a single dose of CP (200 mg/kg dissolved in 2 ml distilled water (DW)); (iii) C_60_ group was given orally of C_60_ (4 mg/kg dissolved in 1 ml VOO) in alternative days for 20 days according to [24, 25]; (iv) CP + C_60_ group was injected i.p. with a single dose of CP (200 mg/kg dissolved in 2 ml DW) and given orally 4 mg/kg dissolved in 1 ml VOO of C_60_ in alternative days for 20 days; and (v) CP + VOO group was injected i.p. with a single dose of CP (200 mg/kg dissolved in 2 ml DW and treated orally with VOO (1 ml in alternative days for 20 days). The experimental animal protocols were carried out by following the guidelines for animal care and were approved by the Ethical Animal Care and Use Committee from the Faculty of Veterinary Medicine, South Valley University, Egypt (application number: VetEg.0465R-2017-2018). After 45 days, the treatments were stopped and rats were left for 24 hours, then three animals from each group were sacrificed. Efforts were maximized to minimize pain suffering of animals. The animals were scarified by cervical dislocation under deep anesthesia using diethyl ether. The whole liver and heart were collected in Carnoy's solution for fixation and frozen at −80°C until used for DNA extraction. Bone marrow aspiration for cytogenetic analysis was performed in all rats.

### 2.2. Chemicals and Fullerene C_60_ Preparation

Cyclophosphamide (Endoxan) was delivered as vials (Baxter Oncology, Halle, Germany), and VOO was commercially purchased (Colavita Extra Virgin Olive Oil Company, New Jersey, USA). Mixture of fullerene C_60_ (99.9% purity) (Shanghai Boyle Chemical Co. Ltd., China) and VOO was prepared according to Baati et al. [24] and Elshater et al. [25] as follows: one gram of C_60_ was dissolved in VOO (200 ml), stirred at ambient temperature in dark for 15 days, and centrifuged (5000 rpm) for 1 h. Using 0.25 *μ*m Millipore filters, the supernatant was filtered through and administered immediately.

### 2.3. DNA Extraction

Carnoy's fixed and frozen liver and heart tissues were used for DNA extraction using QIAamp DNA Mini Kit (Qiagen, Santa Clarita, CA). DNA fragmentation in liver and heart tissues was carried out according to the kit manufacturer's protocol. Briefly, liver or heart tissues (20 mg) were grinded in hypotonic lysis buffer (400 *μ*l; 10 mM Tris base, 1 mM EDTA, and 0.2% Triton X-10), and the cells were centrifuged (10,000 rpm) at 4°C for 5 min. The supernatant (containing small DNA fragments) was mixed with equal volumes of 0.5 M NaCl and absolute isopropyl alcohol for precipitation of DNA. Mixture was stored at −20°C overnight, then centrifuged (11,000 rpm) at 4°C for 15 min. Ethanol (200 *μ*l of 70%) was used for washing the pellet; then, the pellet was loaded into the elution column and washed twice with buffer, and the DNA was isolated using elution buffer. The eluted DNA was stored at −20°C until use [26].

### 2.4. Polymerase Chain Reaction of ISSR Analysis

Five primers (Eurofins, Germany) (Table 1) were used in intersimple sequence repeat (ISSR) analysis, and PCR cycling was performed in a TakaRa Thermal Cycler (Takara Bio Inc., Shiga, Japan). For ISSR analysis, PCR amplification reactions [27] were used in a volume of 25 *μ*l (1X of Green GoTaq® Flexi Buffer, primer (25 pucM), dNTPs (200 *μ*M, Promega), MgCl_2_ (1.5 mM), GoTaq Flexi DNA Polymerase (1 U, Promega), template DNA (25 ng), and up to 25 *μ*l DW). The reactions were carried out in the following conditions: initial denaturation process (1 cycle) was performed at 94°C for 5 min; annealing (40 cycles) was carried out at 94°C for 45 sec, 45°C for 50 sec, and 72°C for 1.5 min; and lastly, extension (1 cycle) was done at 72°C for 7 min.

### 2.5. Agarose Gel Electrophoresis

PCR products were analyzed using agarose gel electrophoresis (1.5%) and visualized with ethidium bromide (10 *μ*g/*μ*l) staining. The gels were exposed to UV light and photographed using a Molecular Imager® Gel Doc™ System with Image Lab™ Software, Bio-Rad. The size of the DNA fragments was estimated based on a DNA ladder (100 to 2000 bp, MBI, Fermentas). The presence or absence of each band was treated as binary character in a data matrix, that is, coded 1 and 0, respectively, The amplification products were scored as (1) for the presence and (0) for the absence of the bands and were compared to the bands in the control group to determine the genetic alterations across the other treated groups. The appearance of new bands and disappearance of existed bands in comparison with the control group are considered DNA polymorphism. Percentage of polymorphism is calculated according to this equation: number of polymorphic DNA band × 100/total number of bands.

### 2.6. Preparation of Chromosome for Chromosomal Aberration Analysis

Microscopic slides for mitotic chromosomal spread were prepared as described by Yosida and Amano [28]. Rats were injected i.p. with colchicines (0.05%) and then euthanized 2 h later. Femurs of rats were removed, and the bone marrow cells were aspirated from both femurs in warmed hypotonic solution (5-6 ml of 0.56% KCl) for 30 min. The aspirate was centrifuged, and the supernatant was decanted. The resultant cellular mass was fixed three times in a mixture of (3 : 1) methanol-glacial acetic acid. Slides were prepared by dropping the cell suspension onto ethanol-cold slides and flaming them slightly. The slides were stained with Giemsa (10%) in phosphate buffer (pH 6.8). In each group, approximately 250 metaphase spreads were analyzed and the structural chromosomal aberrations per cell were counted. Different types of chromosomal aberrations such as chromosomal ring, chromatid deletion, dicentric chromosomes, chromosome fragments, and polyploidy chromosomes were accounted. The data was expressed as chromosomal aberration percentage (%) in each group using the following formula: chromosomal aberration % = total number of chromosomal aberrations × 100/total number of counted metaphase spreads (250).

### 2.7. Statistical Analysis

Statistical analysis was carried out using the Student's *t*-test (two-tailed) with SPSS 24 software. Chromosomal aberrations are expressed as mean ± SE. Differences were considered as significant when *P* < 0.05.

## 3. Results

### 3.1. Genotoxic Changes in Hepatic and Cardiac Tissues

**Figure 1:**
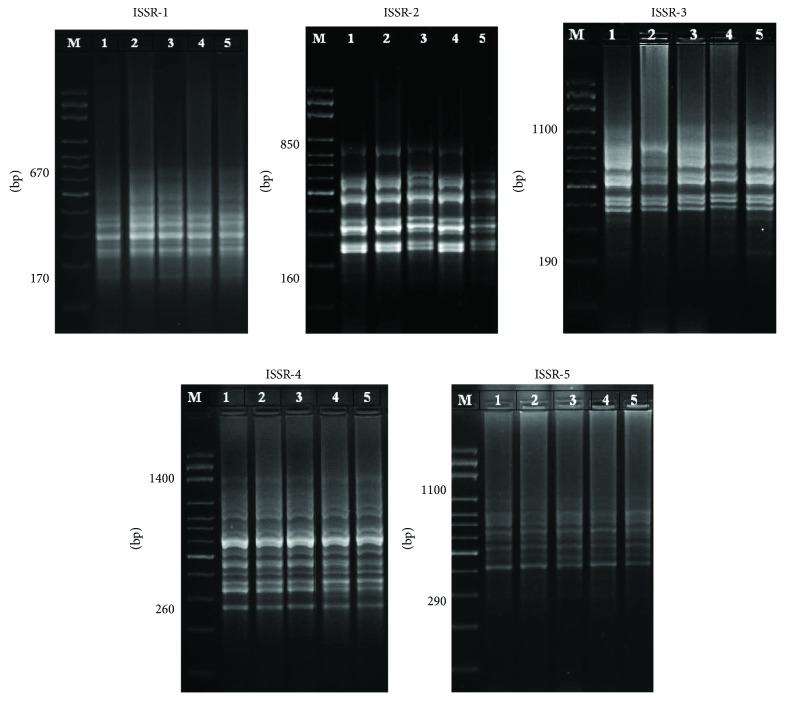
PCR products of liver genomic DNA after treatments with cyclophosphamide, fullerene nanoparticles (C_60_), and virgin olive oil; lane M: DNA marker; lane 1: control; lane 2: CP group; lane 3: C_60_ group; lane 4: CP + C_60_ group; and lane 5: CP + VOO group.

**Figure 2:**
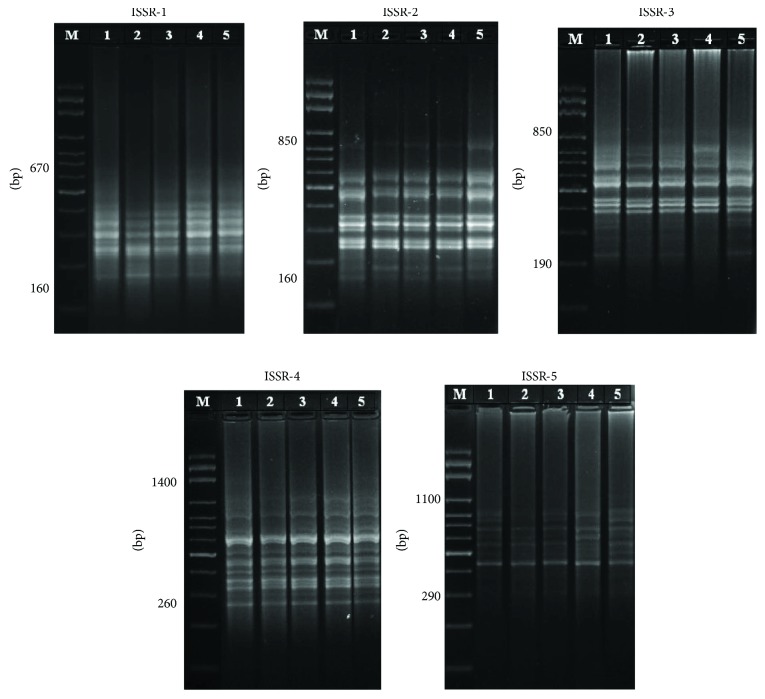
PCR products of heart genomic DNA after treatments with CP, fullerene nanoparticles (C_60_), and virgin olive oil; lane M: DNA marker; lane 1: control; lane 2: CP group; lane 3: C_60_ group; lane 4: CP + C_60_ group; and lane 5: CP + VOO group.

ISSR analysis of the hepatic and cardiac tissues was performed to assess the genotoxicity of CP and the effect of C_60_ and VOO on CP-induced genotoxicity in rats. Numbers of ISSR bands ranged between 8 and 15 and 9 and 17 in hepatic and cardiac tissues, respectively (Figures 1 and 2, Table 2). The amplified bands had molecular weight of genomic DNA ranged between 160 and 1400 bp for both the hepatic and cardiac tissues, with 12 band average per primer (Figures 1 and 2, Table 2). In the control group, there were 57 ISSR bands in liver samples, compared to 52, 57, 56, and 61 bands in CP, C_60_, CP + C_60_, and CP + VOO groups, respectively. The CP group exhibited the highest number of lost bands. The number of lost bands caused by CP was decreased after treatment with C_60_ or VOO (Table 2). CP-induced loss in bands has been improved through treatment with C_60_, as well as VOO.

In cardiac tissues, ISSR bands were 53, 46, 53, 50, and 55 in control, CP, C_60_, CP + C_60_, and CP + VOO groups, respectively (Figure 2, Table 2). Similarly, rats treated with CP had the highest number of ISSR band loss, and treatment with C_60_ or VOO decreased the band loss and recovered to control levels. Treatment with C_60_ and VOO improved the CP-induced genotoxicity. Furthermore, cardiac muscles were more affected by genotoxic effects of CP than hepatic tissues, and the rate of improvement due to treatment with C_60_ was lower than that observed in hepatic tissue.

The highest polymorphic bands were produced by the ISSR-3 and ISSR-1 primers (71.4% and 50% in hepatic and cardiac tissues, resp.) in the CP group (Table 3), whereas the lowest polymorphic bands were produced by ISSR-5 and ISSR-2 (0% and 9.1%) in hepatic and cardiac tissues, respectively (Table 3).

### 3.2. Attenuation of CP-Induced Polymorphism in Hepatic and Cardiac Tissues after Treatment with Fullerene C_60_ and VOO

Genetic analysis revealed that CP induced 11 polymorphic bands out of 52 bands (21.2% polymorphism) detected in the hepatic tissues (Table 3). In cardiac tissues, CP produced 15 polymorphic bands out of 46 bands (32.6% polymorphism) (Table 3). In contrast, the percentages of polymorphism in fullerene C_60_ (7%), CP + C_60_ (8.9%), and CP + VOO (9.8%) were lower than those recorded in CP (21.2%) in liver tissues (Table 3). Band polymorphism in cardiac tissues is recorded in Table 3. The percentages of polymorphism were 32.6, 11.3, 12, and 12.7% in cardiac tissues of CP, C_60_, CP + C_60_, and CP + VOO groups, respectively. Livers and hearts from rats exposed to C_60_ dissolved in VOO (C_60_ group) showed 7% and 11.3% polymorphisms, respectively (Table 3). These results indicate that polymorphisms induced by CP can be attenuated by C_60_ or VOO (in CP + C_60_ and CP + VOO groups).

Comparison of polymorphism percentages in cardiac muscles in all groups with those of hepatic tissues indicated that cardiac muscles (32.6% in CP) were more sensitive to CP genotoxicity than hepatic tissues (21.2% in CP) and the latter was better in response to the effect of C_60_ and VOO in moderation, reduction, or attenuation of CP toxic effects. Moreover, treatment with C_60_ was more effective than with VOO in improving the CP-induced genetic toxicity.

### 3.3. Cyclophosphamide-Induced Chromosomal Aberrations and Effect of C_60_ and VOO

**Figure 3:**
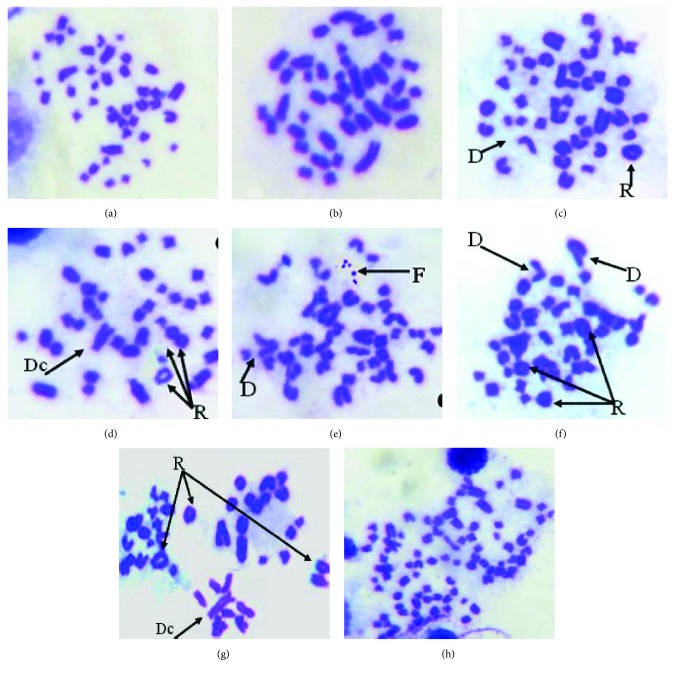
Metaphase-chromosomal aberrations in bone marrow cells showing the effect of CP treatment in group 2; (a and b) normal metaphase. Deletion in chromatid (*D*), ring chromosomes (*R*), dicentric chromosome (*D*_c_), and fragment chromosomes (*F*), and polyploidy in chromosome numbers was observed in (h).

The chromosomal alterations in bone marrow cells due to CP, C_60_, and VOO were recorded. The CP group had the highest level (23.00 ± 14.28) of aberrant chromosomes (Figure 3, Table 4) in comparison to other treated groups. The major chromosomal aberrations were the formation of chromosomal rings (40.0%) (Table 4, Figure 3). Dicentric chromosomes (Figures 3(d) and 3(g)), chromosomal fragments (Figure 3(e)), chromatid deletions (Figures 3(c), 3(e), and 3(f)), and polyploidy were recorded after CP treatment, representing 4.00%, 3.5%, 6.0%, and 4.0%, respectively. Rats cotreated with CP and C_60_ or VOO reduced significantly the percentage of chromosomal aberrations when compared to those with CP alone. The frequencies of total chromosomal aberrations were 0.40 ± 0.24, 23.0 ± 14.28, 8.20 ± 3.29, 10.40 ± 5.10, and 11.20 ± 6.23 in control-, CP-, C_60_-, CP + C_60_-, and CP + VOO-treated animals, respectively. The percentages of total aberrant chromosomes were 1, 57.5, 20.5, 26.0, and 28% in control, CP, C_60_, CP + C_60_, and CP + VOO groups, respectively (Table 4). Phenotype of chromosomal ring was reduced significantly after treatment with C_60_ or VOO (Table 4, Figure 3). Other chromosomal changes such as deletion, dicentric chromosome, fragmentation, and polyploidy were reduced in number and percentage in treatment groups (C_60_, CP + C_60_, and CP + VOO) compared with those in the CP group (Table 4, Figure 3). The total numbers of aberrant chromosomes in 250 bone marrow cells were 2, 115, 41, 52, and 56 in control, CP, C_60_, CP + C_60_, and CP + VOO, respectively (Table 4). C_60_ and VOO significantly reduced chromosomal aberrations (50% reduction or more) in comparison with CP-treated rats.

## 4. Discussion

CP is used as a cancer chemotherapy alkylating agent. Active compounds of CP are acrolein and phosphoramide which are responsible for reducing the growth of cancerous cells by acting at the DNA level [29]. On the other hand, CP can enhance secondary tumors in healthy human tissues such as urinary bladder tumors as well as metastasis occasionally [30, 31]. CP, as a prooxidant, is used for treatment for a long time which leads to oxidative stress through generation of free radicals. After CP treatment, antioxidant enzyme activities decreased and lipid peroxidation increased [32]. Moreover, CP-induced genotoxicity is a dose-dependent manner. Although CP is used as an anticancer drug which is necessary to kill the carcinogenic cells, the increase use of CP in the treatment period leads to cytotoxicity of healthy cells in the body [5]. CP chemotherapy induces a variety of changes in DNA and proteins that lead to imbalance in cell division. In the present study, CP decreased the DNA level and increased the chromosomal aberrations in rat tissues. Most of chemotherapeutic agents and CP cause gene mutations, chromosomal aberrations, and rearrangements in somatic and germ cells of experimental animals [33]. Protection against CP chemotherapy-induced genotoxicity is a hot research point. Many mechanisms have been adopted to deal with CP genotoxicity, and several antimutagens were recorded acting in rodents and may be active in human too [34]. The objective of the current study was to assess the effect of fullerene C_60_ nanoparticles as well as VOO to ameliorate CP-induced genotoxicity. Previous studies were demonstrating an improved effect of some extracts and chemicals such as garlic and *Ocimum sanctum* on the chromosomal aberrations [35, 36]. This study investigated the antigenotoxic activity of fullerene C_60_ as well as VOO in rat tissues by two main methods, molecular assays and cytogenetic biomarkers using DNA fragmentation assay in liver and heart tissues and chromosomal aberrations in bone marrow cells, respectively. CP produced severe mutations in DNA strands and chromosomes giving the ability to fairly judge on the DNA repair and cytogenetic activity of C_60_ and VOO in rats *in vivo*. CP induced loss of ISSR bands that decreased by cotreatment with C_60_ or VOO. CP-induced loss in bands has been improved through treatment with C_60_, as well as VOO. Moreover, treatment with C_60_ was more effective than with VOO in improving the CP-induced genetic toxicity because the percentages of DNA polymorphism in hepatic and cardiac tissues were 8.9% and 9.8% and 12 and 12.7% in CP + C_60_ and CP + VOO groups, respectively. Fullerene C_60_ causes no damage in DNA strands and had no impact on the level of aberrant chromosomes *in vivo* and *in vitro* [15, 37, 38]. In the present study, C_60_ and VOO induced an improved DNA level and decreased the chromosomal aberration in CP + C_60_ or CP + VOO compared to that in the CP group. Fullerene nanoparticles (C_60_) possess an ability to protect against oxidative stress and may decrease mutagenic activity, due to the nanosize [19, 20, 39]. In contrast, few reports stated that C_60_ possesses genotoxic activity in different animal tissues where it induces breaks and oxidative damages of in the DNA strand [12, 13, 40, 41]. In the present study, C_60_ improved the lost ISSR bands in both hepatic (57 and 56 in C_60_ and CP − C_60_, resp.) and cardiac (53 and 50 in C_60_ and CP − C_60_, resp.) tissues when compared to those of CP (52 and 46), respectively. Moreover, C_60_ decreased the percentage of DNA polymorphism in hepatic (7% and 8.9% in C_60_ and CP − C_60_, resp.) and cardiac (11.3% and 12% in C_60_ and CP − C_60_, resp.) tissues when compared to that of CP (21.2% and 32.6%), respectively. Effect of C_60_ on tissues *in vivo* and *in vitro* studies depends on the size of nanoparticles, the given dose, duration of exposure, and type of cells [41, 42]. In the present study, low dose of C_60_ was 4 mg/kg dissolved in VOO as those recorded by Baati et al. [24] and Elshater et al. [25] as low doses are protective against oxidative stress.

Effect of C_60_ on CP-induced hepatotoxicity was more effective than its effect on CP-induced cardiotoxicity and triggered a better response against genotoxicity in the liver than in the heart. Comparison of polymorphism percentages in cardiac muscles in all groups with those of hepatic tissues indicated that cardiac muscles (32.6% in CP) were more sensitive to CP genotoxicity than hepatic tissues (21.2% in CP). Cardiac muscles were worse in response to the effect of C_60_ and VOO than hepatic tissue in moderation, reduction, or attenuation of CP toxic effects.

The chromosomal aberration is an important parameter for investigating the protective effects of antigenotoxic agents on chemical and drug-induced toxicity. C_60_ had no genotoxic effects, and it induced antigenotoxic effects at subcytotoxic concentrations on human lymphocytes, presented by the decreased in micronuclei and chromosomal aberration frequency [37, 38]. Moreover, C_60_ prevents the toxic effect of doxorubicin (chemotherapeutic agent) on normal cells and possesses no genotoxic effect on human lymphocytes [18]. C_60_ has a potential antioxidative effect against CP-induced hepatotoxicity [25]. In the present work, in spite of C_60_ induced few genotoxic features represented by a few number of chromosomal aberration, for example, chromosomal ring, chromosomal fragments, and chromatid deletions, C_60_ had a protective effect against CP-induced genotoxicity. C_60_ as well as VOO decreased the number and types of aberrant chromosomes in CP + C_60_ and CP + VOO groups, respectively, when compared to the treatment by CP alone. Similar findings have been approved by C_60_ and VOO against cadmium-induced genotoxicity [43]. Fullerene C_60_ and VOO significantly ameliorate cadmium chloride-induced genotoxicity in hepatic and renal tissues. Moreover, they reversed the chromosomal alterations caused by cadmium chloride toxicity on bone marrow [43].

Moreover, VOO had an antigenotoxic impact on rat tissues. Our molecular studies on ISSR in hepatic and cardiac tissues as well as cytogenetic of bone marrow cells indicated that VOO alleviated CP-induced genotoxicity. Similarly, Fabiani et al. [22] reported that olive oil has a protective activity against cancer through arrest of the cell cycle and induction of apoptosis in tumor cells and also it has cytotoxic as well as cytoprotective compounds with potential pharmaceutical properties. VOO has a potential antioxidative effect, and it protects DNA from damage induced by a toxic material or a chemotherapeutic agent [21, 44, 45]. This study displays the antioxidant and antigenotoxic activities of fullerene C_60_ nanoparticle and olive oil and its antimutagenic impacts in reducing the DNA damage, which can be occurred in healthy cells as side effect of the treatment with cyclophosphamide.

## 5. Conclusion

The present study investigated the antigenotoxic activity of C_60_ as well as VOO in hepatic and cardiac tissues of a rat after induction of genotoxicity by CP. Two main methods were performed molecular ISSR assay and cytogenetic biomarkers using DNA fragmentation of liver and heart tissues and chromosomal aberrations in bone marrow cells, respectively. CP made severe mutations in DNA strands and chromosomal aberration; in contrast, DNA band numbers return to the control level as well as the chromosomal aberration frequency decreased significantly after C60 and VOO treatments. This study investigated the antigenotoxic activities of C_60_ nanoparticle and VOO and its antimutagenic impacts in reducing the DNA damage in healthy cells (C_60_ group) and after genotoxicity (CP + C_60_ and CP + VOO groups). Virgin olive oil has potent antigenotoxic effect compared with C_60_. Cardiac muscles were more susceptible to CP-induced genotoxicity and less responsive to C_60_ and VOO treatments than hepatic tissues. These findings highlight the principles for the future research possibilities to design and develop C_60_- and VOO-related drugs combined to CP and other chemotherapeutics, which might minimize the side effects caused by the commonly used chemotherapeutic agent.

## Figures and Tables

**Table 1:** The primer code and nucleotide sequences.

Primer	Primer sequence 5′-3′
ISSR-1	5′-ACACACACACACACACYA-3′
ISSR-2	5′-AGAGAGAGAGAGAGAGYT-3′
ISSR-3	5′-CTCCTCCTCCTCCTCTT-3′
ISSR-4	5′-CTCTCTCTCTCTCTCTCG-3′
ISSR-5	5′-TCTCTCTCTCTCTCTCA-3′

Nucleotide code: A = adenine, C = cytosine, G = guanine, T = thymine, and Y = Cor T.

**Table 2:** Number and frequency of the obtained bands using ISSR in liver and heart tissues after treatment with CP, C_60_, and VOO.

Primers	Total band numbers	Mobility range (bp)	Number of bands					Band frequency (mean ± SE)
			Control	CP	C_60_	CP + C_60_	CP + VOO	
*Rat liver*								
ISSR-1	11	170–670	10	11	11	11	11	0.98 ± 0.02
ISSR-2	14	160–850	12	11	14	10	14	0.81 ± 0.07
ISSR-3	13	190–1000	13	7	10	12	13	0.85 ± 0.06
ISSR-4	15	260–1400	14	15	14	15	15	0.97 ± 0.03
ISSR-5	8	290–1000	8	8	8	8	8	1.00 ± 0.00
Sum			**57**	**52**	**57**	**56**	**61**	
*Rat heart*								
ISSR-1	9	160–670	10	10	10	11	11	0.82 ± 0.08
ISSR-2	11	160–850	12	11	11	11	12	0.95 ± 0.05
ISSR-3	11	190–850	12	9	12	7	10	0.83 ± 0.06
ISSR-4	17	260–1400	12	11	13	14	15	0.87 ± 0.06
ISSR-5	11	290–1000	7	5	7	7	**7**	0.83 ± 0.10
Sum			**53**	**46**	**53**	**50**	**55**	

**Table 3:** Detected polymorphism for the ISSR primer in hepatic and cardiac tissues of rats after treatment with CP, C_60_, and VOO.

Primer	CP			C_60_			CP + C_60_			CP + VOO		
	Bands (number)	Polyploidy (number)	Polymorphism (%)	Bands (number)	Polyploidy (number)	Polymorphism (%)	Bands (number)	Polyploidy (number)	Polymorphism (%)	Bands (number)	Polyploidy (number)	Polymorphism (%)
*Liver*												
ISSR-1	11	1	9.1	11	0	0	11	0	0	11	1	9.1
ISSR-2	11	4	36.4	14	0	0	10	4	40	14	4	28.6
ISSR-3	7	5	71.4	10	3	30	12	1	8.3	13	0	0
ISSR-4	15	1	6.7	14	1	7.1	15	0	0	15	1	6.7
ISSR-5	8	0	0	8	0	0	8	0	0	8	0	0
Sum	52	11	21.2	57	4	7	56	5	8.9	61	6	9.8
*Heart*												
ISSR-1	10	5	50	10	1	10	11	0	0	11	0	0
ISSR-2	11	1	9.1	11	1	9.1	11	1	9.1	12	1	8.3
ISSR-3	9	3	33.3	12	2	16.7	7	3	42.9	10	2	20
ISSR-4	11	4	36.4	13	2	15.4	14	1	7.1	15	3	20
ISSR-5	5	2	40	7	0	0	7	1	14.3	7	1	14.3
Sum	46	15	32.6	53	6	11.3	50	6	12	55	7	12.7

**Table 4:** Chromosomal aberrations in rat bone marrow cells after treatment with CP, C_60_, and VOO.

Groups	Aberration cells (number)	Chromosomal aberration					Total aberrations		Average number of aberration (mean ± SEM)
		*R* (number)	*D* (number)	*D*_c_ (number)	*F* (number)	Poly (number)	Number	%	
Control	2	1	1	—	—		2	1.00	0.40 ± 0.24
CP	95	80	12	8	7	8	115	57.5	23.00 ± 14.28^∗^
C_60_	25	20	10	6	1	4	41	20.5	8.20 ± 3.29^∗^
CP + C_60_	36	30	10	7	3	2	52	26.0	10.40 ± 5.10^∗^
CP + VOO	45	36	7	3	5	5	56	28.0	11.20 ± 6.23^∗^

*R* = ring chromosome, *D* = deletion in chromatid, *D*_c_ = dicentric chromosome, *F* = fragmentation chromosomes, and Poly = polyploidy chromosomes. Values are mean of replicates ± SEM. ^∗^Significant at *P* < 0.05.

## Data Availability

The data used to support the findings of this study are available from the corresponding author upon request.
